# A thematic analysis of alcohol use and culture amongst elite (intercounty) Gaelic Athletic Association (GAA) players

**DOI:** 10.1007/s11845-023-03394-7

**Published:** 2023-05-08

**Authors:** Conall Mac Gearailt, Colm Murphy, Jack McCaffrey, Matthew Turk, Kieran Murray

**Affiliations:** 1 University Hospital Limerick and University of Limerick, Limerick, Ireland; 2 Washington Street Medical Centre, Cork, Ireland; 3 Children’s Health Ireland, Temple Street, Dublin, Ireland; 4 University College Dublin, Dublin, Ireland

**Keywords:** Alcohol, Binge drinking, GAA, Thematic analysis

## Abstract

**Background:**

There are limited studies examining alcohol consumption in Gaelic Athletic Association (GAA) players. In a previous paper, we reported excess alcohol consumption, alcohol-related harms and binge drinking amongst elite GAA players. In that survey, the players were provided with an opportunity to provide comments on alcohol. This current study analyses these comments.

**Aims:**

The aim of this study was to provide a qualitative analysis of elite GAA players opinions on alcohol consumption, harms, behaviours and culture.

**Methods:**

An anonymous, web-based e-questionnaire was distributed to all registered adult elite (inter-county) GAA players. This analysed demographics, alcohol consumption, alcohol culture and alcohol-related harms. This paper is a thematic analysis of the players comments on alcohol in the GAA.

**Results:**

Seven hundred seventy-three of 3592 (21%) players responded. One hundred fifty-two respondents (21%) commented in the free text section of the survey regarding alcohol. One hundred eleven comments (73%) were suitable for analysis. Relevant themes were a pattern of abstinence and bingeing (*n* = 44), excess alcohol consumption (*n* = 40) and drinking bans contributing to a binge drinking culture (*n* = 37). There was a mixed attitude to alcohol sponsorship.

**Conclusion:**

These data show players recognise intermittent binge drinking with periods of abstinence and alcohol-related harms. Further initiatives regarding alcohol harm reduction merit consideration including prohibition of alcohol sponsorship, similar to the GAA’s ban on gambling.

## Introduction

Alcohol is a significant source of health harm, to the drinker, their friends and family and to society at large. Increased alcohol intake is associated with increased mortality [1]. Alcohol is the third highest preventable cause of death and is involved in approximately 5% of deaths worldwide [2, 3]. Despite prior suspicion of benefit in small amounts, newer extensive epidemiological evidence is suggestive that there is no evidence of benefit of drinking alcohol on cardiovascular or on all-cause mortality [4, 5]. It is now clear that there is no level of alcohol that improves overall health [6]. Binge drinking is considered drinking six or more standard drinks in one sitting [7]. Binge drinking is associated with significant risks including an increased morbidity and mortality [8]. Morbidity can entail episodes such as fights, falls, road traffic accidents as well as suicidality. In an Irish cohort, a younger population was more likely to binge drink with 52% of people under 35 drinking in this way, whereas only 21% of those over 65 would do so [7].

The Gaelic Athletic Association (GAA) is an Irish amateur sporting organisation founded in 1884. It is focused on the forwarding of native Irish sports such as Gaelic football, hurling, handball and rounders in Ireland and abroad. The organisation has both male and female participants [9]. Gaelic football and hurling are the most popular field sports in Ireland in terms of supporter attendances at elite level with roughly 1.5 million people attending the elite (inter-county) championships annually [10]. The Gaelic Players Association represent elite male and female athletes compromising of over 4000 members having recently merged with the Women’s Gaelic Players Association (WGPA) [11].

## Methods

### Design

A point prevalence study was performed to analyse the alcohol consumption and alcohol behaviours and gambling culture amongst elite GAA players. The 32-question questionnaire was part of a larger 61 question study entitled *Alcohol and Gambling in Inter-county GAA Players*. It involved quantitative and qualitive components and is described in detail in a prior paper in this journal and another [12, 13].

A thematic analysis can help focus on themes and behavioural patterns to develop a comprehensive understanding of the data [14]. To examine for factors influencing alcohol consumption and behaviours in survey participants, a thematic analysis was performed of the responses to the free text section of the survey under the heading: *Please write here any additional comments you have about alcohol in the GAA.* This paper analyses these comments.

## Results

**Fig. 1:**
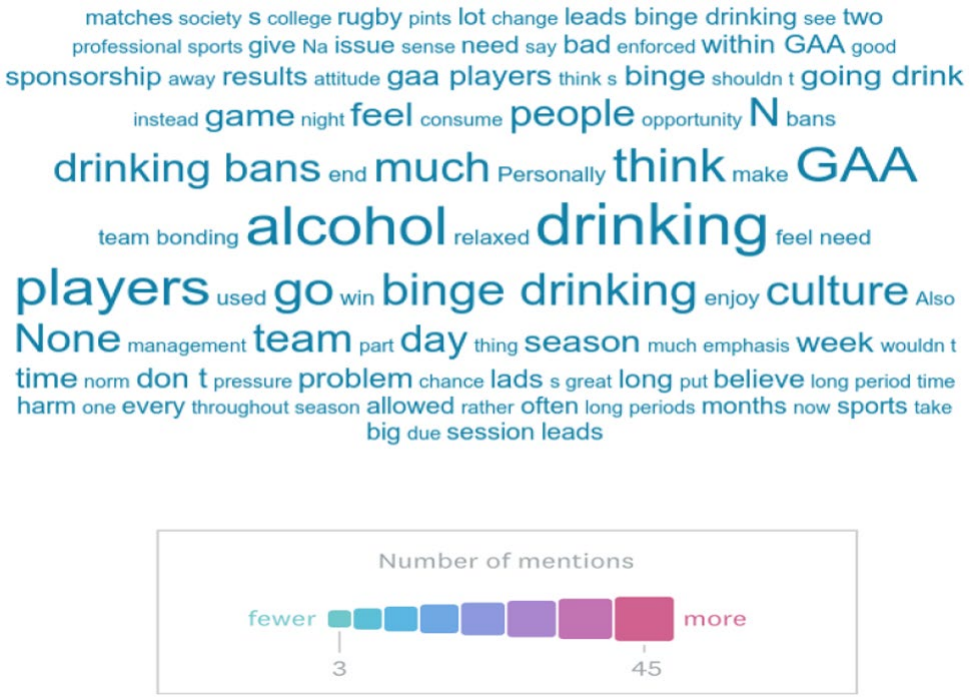
Word cloud comments on alcohol in the GAA

One hundred fifty-two (21%) respondents commented in the free text section of the survey entitled “*Please write here any additional comments you have about alcohol in the GAA*”*.* A word cloud was created (Fig. 1) and a thematic analysis of the responses was performed.

Forty-one comments did not provide any feedback (None/N/a/. etc.) and were subsequently discounted, leaving 111 comments suitable for analysis. The major themes were reported in order of their frequency and one or two representative comments from each. Many comments covered multiple themes.

### On versus off mentality (n = 44)

Many respondents felt players are unable to drink for long periods for numerous reasons (drinking bans, peer pressure, inability to perform in their chosen sport) and then drink heavily when the opportunity arises:

"In the GAA there seems to be a culture of ‘full duck or no dinner’. What I mean by this is players tend to abstain from alcohol for long periods of time and then when they do drink they binge heavily. I am definitely guilty of this."

### Binge drinking (n = 40)

There was a clear overall message in the responses that the culture in elite GAA is of drinking to excess as opposed to in moderation. Some respondents suggested that due to being unable to drink in moderation throughout the year, they over-indulge in the off season.

"People drink to excess when the opportunity arises because most of the season you can’t because of training etc."

### Drinking ban (n = 34)

Drinking bans were a frequent topic of discussion with the feedback being overwhelmingly negative (97%). It was felt they create a culture of binge drinking when the ban lifted and can create mistrust amongst teammates and management.

"Drinking bans are detrimental. They encourage binge drinking insofar as they stop you from drinking for a long period of time and so you feel obliged to get as much out of a drinking session as you can."

### Elite GAA and alcohol heavily intertwined (n = 17)

During the season, players spend long periods together and often have limited social outlets. At the conclusion of competitions, drinking seems to be an integral part of celebration of victory or commiseration of defeat.

"I believe players use alcohol to bond as a team as well as celebrate victories. Since us as G.A.A players are restricted to drink for long periods of times we seem to drink a lot more alcohol than usual to make up for it."

### Irish society in general (n = 16)

A number of respondents felt the issues were not so much related to the GAA, but to external factors such as the age of players and Irish society in general. Two respondents commented on this being a “college” issue.

"Purely a society issue."

### Learn from other sports (n = 13)

Thirteen respondents highlighted a preferable attitude to alcohol in other sports (rugby/soccer/Australian rules football). There was felt to be more frequent, more moderate drinking in these sports. Some questioned the sacrifices expected of the amateur GAA players with prolonged drinking bans, compared to the professional athletes above. In particular, rugby was felt to have a healthier approach and was mentioned seven times.

"Professional athletes go for their few drinks whenever they want nothing said! When an amateur athlete that gives up his or her free time go for a few drinks, they are blackened by the GAA community!! Doesn’t make much sense does it."

### Alcohol sponsorship of GAA (n = 12)

There was a mixed attitude to alcohol sponsorship and promotion. Five expressed positive opinions, six negative and one neutral. One respondent mistakenly believed alcohol sponsorship is prohibited by the GAA. Perhaps, this confusion is related to the recent ban on gambling sponsorship.

"No harm to get sponsorship money from them. Get something back."

"It was an important step to ban alcohol sponsorship and that must be maintained."

### Alcohol in GAA not an issue (n = 8)

Some participants felt that this topic is exaggerated.

"No issue with alcohol in the GAA."

### Potential for positive effects (n = 7)

The potential role of alcohol in increasing team cohesiveness and enabling players to “blow off some steam” was highlighted.

"We’re amateur athletes at the end of the day so there shouldn’t be so much of a bad stigma about county players going out for a drink plus I think there’s a lot to be said for teammates going out and drinking together it’s great for team bonding."

### Elite GAA detrimental to social life (n = 6)

Some respondents feel a pressure to avoid socialising in general during the season as even to be seen out socialising during this period can be negatively perceived:

"I feel it effects players social lives very much. Even if you are seen out not drinking you are presumed to have been drinking which is unfair."

### Alcohol’s effect on sporting performance (n = 5)

A number of players report choosing themselves to abstain from alcohol or drink less as they feel it is detrimental to their playing form:

"I personally can’t…drink and perform on the pitch and in college so it’s one or the other for me. I usually keep drinking alcohol to big events rather than finding myself falling into a routine of going out every week. Players can’t expect to improve if they are consistently drinking and it’s up to them to realise."

### Alcohol and GAA linked from an early age (n = 2)

Some respondents feel that underage GAA members grow up associating the sports with alcohol:

"It is becoming increasingly common in our underage level with clubs to choose to go back and celebrate, host awards nights, fundraisers, etc. in the local…certain parents or coaches want to go to pubs instead which leads to all the underage members of our club growing up in the same drinking culture and heavily associating it with GAA. There are also many occurrences of underage drinking as a result. I have seen children as young as 11 or 12 drinking after county final celebrations."

### Alcohol culture is improving (n = 2)

Some felt the culture is changing for the better.

"I do not know if there is a wider problem but within my own camp there does not appear to be the same level of consumption or frequency of consumption as there was 10 years ago."

### Individuals differ (n = 2)

"Depends on the person personality/addictiveness."

## Discussion

This study gives a new insight into the drinking culture of elite GAA players both male and female. It is one of the first studies in which an analysis of alcohol culture is performed in GAA. Here, we examine the culture related to alcohol in an elite sporting cohort using their own words and expressions. It is also a rare study in which alcohol-related behaviours are examined in female GAA players. Female athletes are frequently neglected in studies, and this study addresses this unmet need.

The sample size of respondents in the study is a strength with more than three times the participants as a previous mental health study [10]. All registered players were contacted to participate in this study avoiding bias. The self-reported nature of the survey could be regarded as a weakness. However, these have been previously validated in the study of alcohol behaviours [15, 16].

The thematic analysis raised some key terms unprompted. The most common terms used included “alcohol”, “drinking”, “binge”, “drinking bans” and “culture”. This suggests that a culture of drinking and indeed that binge drinking is to the forefront of players’ minds when asked to comment on alcohol and the GAA. Studies of other elite sports suggest a higher rate of binge drinking compared to their peers [17]. Some players postulate that this may be due to the all or nothing aspect of elite sports. They feel that they cannot engage fully in a regular social life and subsequently overindulge when given an opportunity. Many felt that drinking bans have a counter-productive effect and lead to bingeing as mentioned previously.

The mixed attitude towards alcohol sponsorship in the GAA was interesting. A conservative estimate of the value of alcohol sponsorship to Irish sport was approximately €35 million [18]. Players opinion of this sponsorship was split equally between positive and negative. Notably, 48% of respondents to our survey reported alcohol sponsorship over the past year. This could lead to a conflict of interest amongst these elite players.

## Conclusions

Similar to US data, participants described higher rates of binge-drinking and increased quantity and frequency of alcohol consumption compared with their peers [19, 20]. This study also demonstrates the idiosyncratic seasonal alcohol consumption pattern within the GAA. Alcohol intake was higher in the off season than during the season. This would tally with Australian data on elite athletes [21].

One respondent reported a “toxic drinking culture” in elite GAA. Given the data in this survey shows excess alcohol consumption, alcohol-related harms and binge drinking, this would seem an accurate assessment. These findings replicate prior studies of both elite and non-elite male GAA players [10, 22, 23]. Interestingly, the gambling section of this survey showed problem gambling rates of 6%, far in excess of the population baseline [13]. In multivariate analysis, having gambled in the past year had an odds ratio of 2.3 (95% confidence interval 1.3–3.9) for adverse alcohol use (AUDIT-C ≥ 5) [12].

The ill effects of alcohol intake are numerous and are well documented [24]. Furthermore, problems with alcohol persist in both the GAA and broader Irish society and indeed worldwide. Like many field sports, Gaelic games are intricately linked with alcohol at all standards. This extends from local public houses sponsoring small rural GAA clubs to the naming of a prominent Dublin nightclub during the 2011 All Ireland inter-county football Championship victory speech.

A harmful drinking culture exists within elite GAA. It requires a varied approach involving grassroots GAA members, the GPA as well as governmental initiatives to combat its effect. Community-based interventions have been shown to alter public opinion on issues such as alcohol [25]. On a national level, reducing alcohol affordability through taxation or price regulation is the most effective and cost-effective way to reduce alcohol-related harms [26]. Indeed, the data on minimum unit pricing (MUP) in Scotland and Wales appears to be promising with limited data on the recent Irish implementation of the same policy [27].

One potential area of intervention is banning alcohol sponsorship of GAA competitions, teams and events. Alcohol sponsorship is associated with an increased risk of hazardous drinking [28]. The GAA is a community-based volunteer organisation with the stated aim of promoting Gaelic games, culture and lifelong participation, and describes player welfare as a core value [29]. The organisation has previously shown a commitment to prioritising the health of members over profit. In 2018, sponsorship of GAA events or activities by gambling firms was prohibited following a motion in GAA congress [30]. A similar ban on alcohol sponsorship as gambling may have a positive effect on alcohol misuse in the GAA setting.

## Data Availability

The data that support the findings of this study are available from the corresponding author upon reasonable request.
